# *Phaseolus vulgaris* L. var. Venanzio Grown in Tuscany: Chemical Composition and In Vitro Investigation of Potential Effects on Colorectal Cancer

**DOI:** 10.3390/antiox9121181

**Published:** 2020-11-26

**Authors:** Federica Finetti, Marco Biagi, Jasmine Ercoli, Giulia Macrì, Elisabetta Miraldi, Lorenza Trabalzini

**Affiliations:** 1 Department of Biotechnology, Chemistry and Pharmacy, University of Siena, Via Aldo Moro 2, 53100 Siena, Italy; ercoli3@student.unisi.it (J.E.); giulia.macri@student.unisi.it (G.M.); 2 Department of Physical Sciences, Earth and Environment, University of Siena, Via Laterina 8, 53100 Siena, Italy; marco.biagi@unisi.it (M.B.); elisabetta.miraldi@unisi.it (E.M.)

**Keywords:** *Phaseulus vulgaris* L., biodiversity, nutraceuticals, oxidative stress, inflammation, functional food, COX-2, colon cancer

## Abstract

*Phaseolus vulgaris* L. (common bean) is a leguminous species that is an important dietary component due to its high content of proteins, unsaturated fatty acids, minerals, dietary fibers and vitamins. Due to the high content of polyphenols, several biological activities have been described for bean extracts, making it possible to include *P. vulgaris* among food with beneficial effects for human health. Moreover, more than 40,000 varieties of beans have been recognised with different nutraceutical properties, pointing out the importance of food biodiversity. In this work, we describe for the first time the chemical composition and biological activity of a newly recognized Italian variety of *P. vulgaris* grown in a restricted area of the Tuscany region and named “Fagiola di Venanzio”. Fagiola di Venanzio water extract is rich in proteins, sugars and polyphenols and displays antioxidant, anti-inflammatory and antiproliferative activities in *in vitro* assays on colon cancer cellular models. Our data indicate that this variety of *P. vulgaris* appears to be a promising source of bioactive compounds and encourage more in-depth studies to better elucidate the implications of its consumption for public health.

## 1. Introduction

Biodiversity and dietary habits are crucially important to prevent the development of lifestyle-associated diseases. In the last few years, there has been an increased interest in the consumption of bioactive foods, capable of exerting biological effects at different cellular levels and endowed with beneficial properties for public health [1]. *Phaseolus vulgaris* L. (common bean) is the most important edible legume in the diet and gastronomy of many countries in the world. Numerous species of *Phaseolus* are cultivated and more than 12 million tons of dry beans are produced worldwide [2]. *P. vulgaris* exists in many variations regarding growth characteristics, maturation and adaptation, accounting for more than 40,000 varieties. Common beans play an important role in human nutrition as a fundamental source of plant proteins, unsaturated fatty acids, minerals, dietary fibers and vitamins [3,4]. Moreover, in the past years beneficial effects for human health have been described and have been associated with the high content of phenolic compounds. In the majority of characterized bean extracts, the phenolic content is mainly represented by phenolic acids, hydroxycinnamic acids, flavones, flavanols, flavanones, isoflavonoids, anthocyanins, chalcones and dihydrochalcones [4,5].

The consumption of beans have received increased attention due to their beneficial health effects in the prevention and control of numerous chronic and degenerative diseases, that are the main causes of mortality in the world. In particular, beans consumption has proven to be effective in reducing the risk of cardiovascular diseases (for the antioxidant, anti-inflammatory and hypolipidemic properties), obesity and diabetes (for the presence of α-amilase inhibitors and phytohemagglutinin and for the presence of starch) and cancer [2,4,6,7].

Several epidemiological studies suggest a link between a diet rich in beans and reduced risk of numerous types of cancer. Beans consumption for two or more times per week reduced the risk of colon cancer (up to 47%) [8] and prostate cancer (about 22%) [9]. These data were also confirmed in animal models in which a diet rich in beans reduced the incidence of tumors as colon cancer [3,10,11,12,13,14,15,16] and breast cancer [17,18] by interfering with multiple signaling pathways. Moreover, in vitro cellular models showed that bean extracts exert antiproliferative, anti-inflammatory, and pro-apoptotic activities in different types of cancer cells [3,13,14,19].

**Figure 1:**
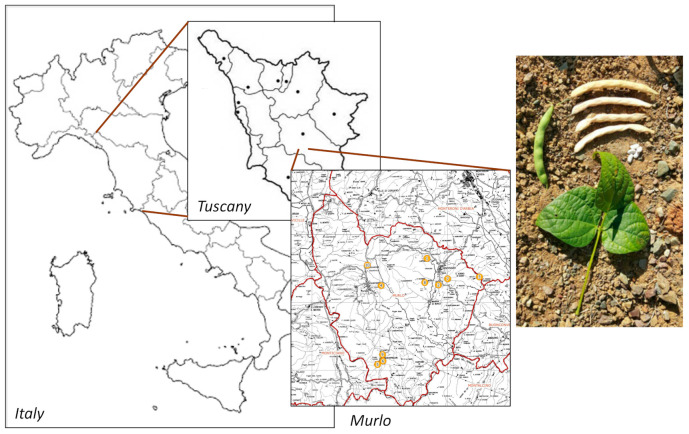
Production area of Fagiola di Venanzio (FV). Colored circles indicate the micro areas within Murlo municipality where FV is cultivated.

In this work, we studied for the first time an endangered Italian variety of *P. vulgaris*, grown in a restricted area of the municipality of Murlo (Siena, Tuscany) named “Fagiola di Venanzio” (FV) (Figure 1). FV has been cultivated since the mid-nineteenth century by the Burresi family in their farm located near Murlo and has been recognized as a specific variety in 2017 (N. VE_145 20-12-2017, Regione Toscana, Italy). FV beans are characterized by a white seed coat color, with a relatively small size, very flattened and elliptical-wide shape (Figure 1). Here, we first determined the chemical composition of FV extracts and then we studied antioxidant, anti-inflammatory and antiproliferative activity on colorectal cancer cellular models.

## 2. Material and Methods

### 2.1. Preparation of Extracts of FV

*Phaseolus vulgaris* L. var. “Fagiola di Venanzio” (FV) dried seeds (beans), were harvested in the municipality of Murlo (Siena, Tuscany, Italy, Latitude 43°10′16″32 N, longitude 11°23′32″28 E) and were identified by botanists in the Siena University Botanical Garden. Four samples of FV provided by different growers were used (Table 1).

In order to preserve the whole phytocomplex of beans and to extract polyphenols, proteins and carbohydrates at the same time, the extractive procedure was accomplished by briefly soaking 10 g of manually grinded beans in water at 50 °C, discarding the liquid and then performing a maceration at 35 °C with 100 mL of distilled water for 48 h. The extract was adjusted to a 1:10 final drug:extract ratio.

### 2.2. Polyphenols Content

Total polyphenols content (TPP) of FV extracts was evaluated by Folin–Ciocalteau (FC) colorimetric assay, optimizing the procedure reported in Biagi et al., 2019 [20]. Briefly, 100 µL of extract was diluted to 3 mL with distilled water; 500 µL of 1:10 FC reactive in water (Sigma-Aldrich, Milan, Italy) were added and the mixture was gently shaken for 1 min. A quantity of 1000 µL of 30% *w*/*v* sodium carbonate water solution was added and, after incubation for 1 h in the dark at RT, absorbance of samples was read at 750 nm, using distilled water as blank. Gallic acid (Sigma-Aldrich) was used as reference standard. A calibration curve was created using gallic acid 5000 to 78 mg/L.

### 2.3. Soluble Carbohydrates Content

Total soluble carbohydrates of extract were quantified using the acid phenol assay described for the first time by Dubois et al., 1951 [21], with optimization of the method. A quantity of 100 µL of the supernatant was added to 190 µL of water and 100 µL of a 6% *w*/*v* phenol (Sigma-Aldrich) water solution. The solution was gently shaken for 30 s and 500 µL of concentrated sulfuric acid (Sigma-Aldrich) were added. The mixture was heated at 80 °C for 15 min and cooled to RT. Absorbance was read at 490 nm. D-glucose (Sigma-Aldrich) was used from 80 to 1.25 mg/L as reference standard. Total saccharide content was calculated, interpolating the data on the calibration curve of D-glucose.

### 2.4. Protein Content

Total proteins of extracts were determined spectrophotometrically using the BCA protein assay kit (Euroclone, Milan, Italy). Briefly, 2 µL of different dilutions of FV extract were added to 100 µL of bicinchoninic acid and, after incubation at 37 °C for 30 min, the absorbance was measured at 562 nm with a microplate reader (EnVision, PerkinElmer, Waltham, MA, USA). Protein concentration was determined and reported with reference to standards of a bovine serum albumin (BSA).

### 2.5. HPLC-DAD Analysis on Main Polyphenolic Constituents

HPLC-DAD analysis was performed by using a Shimadzu Prominence LC 2030 3D instrument equipped with a Bondapak^®^ C18 column, 10 µm, 125 Å, 3.9 mm × 300 mm column (Waters Corporation, Milford, MA, USA).

Water solutions containing 0.1 % (*v*/*v*) formic acid (A) and 0.1% (*v*/*v*) acetonitrile (B) were used as mobile phase. The following program was applied: B from 10% at 0 min to 35% at 20 min, then B 50% at 25 min; flux was set at 0.8 mL/min. Chromatograms were recorded at 254, 280, 330 and 350 nm. Analyses were performed using 10 µL of FV extract; gallic acid, chlorogenic acid, caffeic acid, catechin, genistein, daidzein, quercetin and kaempferol (Sigma-Aldrich) were used as external standards. Calibration curves were established using reference standards ranging from 0.008 mg/mL to 0.500 mg/mL. The correlation coefficient (R^2^) of each curve was >0.99.

### 2.6. HPLC-DAD-DPPH (2,2-Diphenyl-1-picrylhydrazyl)

To evaluate the different role of FV polyphenols in exerting antiradical activity, the HPLC-DAD run described above was repeated after having incubated the FV extract with a 1 × 10^−2^ M DPPH (2,2-diphenyl-1-picrylhydrazyl) methanolic solution for 15 min. Each chromatogram peak area was compared before and after DPPH reaction. Ascorbic acid was used to validate the test.

### 2.7. Cell Culture

HCT116, colorectal carcinoma cells, (ATCC, Rockville, MD, USA) were cultured in DMEM (Euroclone) supplemented with 10% fetal bovine serum (FBS, Euroclone), 100 U/mL penicillin/streptomycin (Euroclone), and 4 mM L-glutamine (Euroclone).

HT29, colorectal adenocarcinoma cells, (ATCC, Rockville, MD, USA) were cultured in RPMI-1640 (Euroclone) medium supplemented with 10% FBS with 100 U/mL penicillin/streptomycin. Both cell lines were grown at 37 °C and 5% CO_2_.

Human Umbilical Vein Endothelial Cells (HUVECs) were purchased from Lonza (Lonza, Basel, Switzerland). All experiments were performed on low passage cell cultures. Cells were grown on gelatin-coated dishes in Endothelial Growth Medium (EGM-2) (EBM-2, FBS 10%, VEGF, R3-IGF-1, hEGF, hFGF, hydrocortisone, ascorbic acid, heparin and GA-1000) (Lonza) at 37 °C and 5% CO_2_.

### 2.8. MTT Assay

Either 3.5 × 10^3^ (HT29) or 2.5 × 10^3^ (HCT116) cells/well were seeded in 96-multiwell plates in medium with 10% FBS and, after adherence, were maintained for 18 h in medium without phenol red (0.1% serum) with different concentrations of the FV extract (5, 10, 50 and 100 µM) and then exposed to IL1β (10 ng/mL) for 24 h.

After 24 h, the medium was removed, and cells were incubated for 4 h with fresh medium in the presence of 1.2 mM MTT (3-(4,5-dimethylthiazol-2-yl)-2,5-diphenyltetrazolium bromide) (Sigma-Aldrich). The MTT solution was then removed and 100 µL of DMSO were added to each well to dissolve the blue formazan crystals. The absorbance of the formazan dye was measured at 570 nm with a microplate reader (EnVision, PerkinElmer, Waltham, MA, USA). Data were expressed as a percentage of the basal control [22].

### 2.9. Western Blotting Analysis

HT29 or HUVEC (3.0 × 10^5^ cells/well) were seeded in 6-well multiplates in medium added with 10% serum. After 24h cells were starved for 6 h in medium containing 0.1% serum, and then were treated with extracts for 18 h (10 and 50 µM) and then exposed to IL1β (10 ng/mL). After 24 h, extraction of total proteins was performed by lysing cells in precooled radioimmunoprecipitation assay (RIPA) lysis buffer.

Protein concentration of cell extracts was determined spectrophotometrically using the BCA protein assay kit (Euroclone). For western blotting analysis, aliquots of cell extract supernatants containing an equal amount of proteins (50 μg) were treated with Laemmli buffer, boiled for 10 min, resolved on 4–20% stain-free gel and then blotted onto a nitrocellulose membrane using Semidry Electro-blotter System (Galileo Bioscience, Cambridge, MA, USA). To determine glutathionylated proteins western blotting of total extract was performed under non reducing conditions. The blots were blocked with 5% defatted dry milk (Euroclone) in tris-buffered saline (TBS) containing 0.5% tween 20 for 1 h, at RT, and incubated overnight at 4 °C with appropriate dilutions of primary antibodies. Subsequently, membranes were incubated for 1h with horseradish peroxidase (HRP)-conjugated secondary antibodies. Proteins were then visualized by an enhanced chemiluminescence detection system (Euroclone). The primary antibodies used in the present study included anti-COX-2 (Cell Signaling Technology, Leiden, The Netherlands), anti-p-ERK1/2 (Cell Signaling Technology), p-NFkB (Cell signaling Technology), anti-GSH (Virogen, Watertown, MA, USA), and anti-GAPDH (EMD Millipore, Darmstadt, Germany). Affinity-purified HRP-conjugated secondary antibodies were from Sigma-Aldrich. Protein bands from western blots were quantified by densitometry using the ImageJ software, and their relative amounts were normalized to the levels of housekeeping proteins serving as internal loading controls [23].

### 2.10. ROS Measurement

ROS levels were evaluated as previously reported [24]. 3.5 or 2.5 × 10^3^ cell/well (HT29, HCT116 or HUVEC) were seeded in 96-multiwell plates and, after adherence, were maintained for 18 h in medium without phenol red (0.1% serum) with different concentrations of the extract (10 and 50 µM) and then exposed to IL1β (10 ng/mL) for 24 h. DCFH_2_-DA (2,-7-dichlorodihydrofluorescein diacetate) (Invitrogen, Milan, Italy) was added (10 µM, 1h) and intracellular levels of ROS were evaluated photometrically with a microplate reader (excitation/emission 495/527) (EnVision, PerkinElmer).

### 2.11. Statistical Analysis

Data were generated from three independent experiments and expressed as mean ± standard deviation (SD). Statistical analysis was performed using Student’s t test for unpaired data; differences in dataset with *p* < 0.05 were considered statistically significant.

## 3. Results

### 3.1. Chemical Composition of FV Extracts

Chemical analyses of FV extracts obtained from four different samples provided by different growers (Table 1) were performed. To investigate the polyphenolic fraction of FV extracts and to identify the main chemical constituents, a high-performance liquid chromatography-diode array detection (HPLC-DAD) analysis was carried out. FV was found to be rich in polyphenols (Table 2), in accordance with other published papers on the species [4].

**Figure 2:**
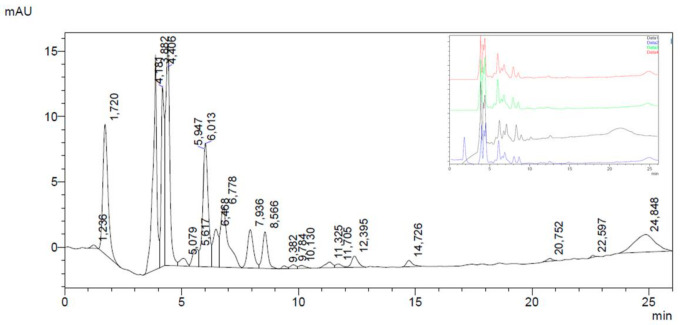
HPLC-DAD profile of polyphenols present in FV extract. Chromatogram of sample #1. In the top box, chromatograms of all samples are reported: blue = sample #1, grey = sample #2, green = sample #3 and red = sample #4.

HPLC-DAD analysis revealed that two main polyphenols subclasses could be identified in FV extracts, namely simple phenolic acids and hydroxycinnamic derivatives. As a water extraction was performed, the prevalence of hydrophilic compounds in the extract was expected and consistent with a previous work on common beans endemic of Southern Italy [3]. Gallic acid (Figure 2 related to #1 FV extract, Retention Time, RT = 4.41 min) and chlorogenic acid (RT = 7.94 min.) resulted the main phenolic and hydroxycinnamic acid, respectively. The other main peaks before gallic acid (RT = 3.88–4.18 min) could be assigned to phenolic acids by monitoring UV spectra for their typical λmax at 270–280 nm. Other hydroxycinnamic derivatives, different from caffeic acid, were recognized by UV spectra (λmax at 270–280 nm and 320–330 nm) and linked with the peak at 6.47 min. Flavonoids were found only in small amounts. At RT = 11.33, 11.71 and 12.40 min, the zone of the chromatogram where isoflavones are recorded, two constituents with UV spectrum similar to genistein and daidzein (λmax at 250–255 nm), but with different RTs, were found. Other flavonoids referable to used standards and their derivatives were not detected as present in concentrations below the detection limits of the method. Table 3 shows the quantification of gallic and chlorogenic acid identified in FV extracts and total phenolic and hydroxycinnamic derivatives, expressed as gallic and chlorogenic acid, respectively.

Due to the minimum variance in polyphenols content and a strong similarity in chromatogram profiles of the four different samples (see Table 2 and Figure 2), only the extract obtained from sample #1, available in highest amount, was further analyzed and investigated in chemical and biological tests. Table 3 summarizes the chemical composition of the selected FV extract of sample #1. In accordance with known nutritional data and literature on *P. vulgaris* (nutritiondata.com), carbohydrates represent the main class of metabolites: the concentration of soluble carbohydrates in the FV extract was shown to be 10.032 mg/g. The concentration of total proteins was 15.190 mg/g.

### 3.2. DPPH Test and HPLC-DAD-DPPH

**Figure 3:**
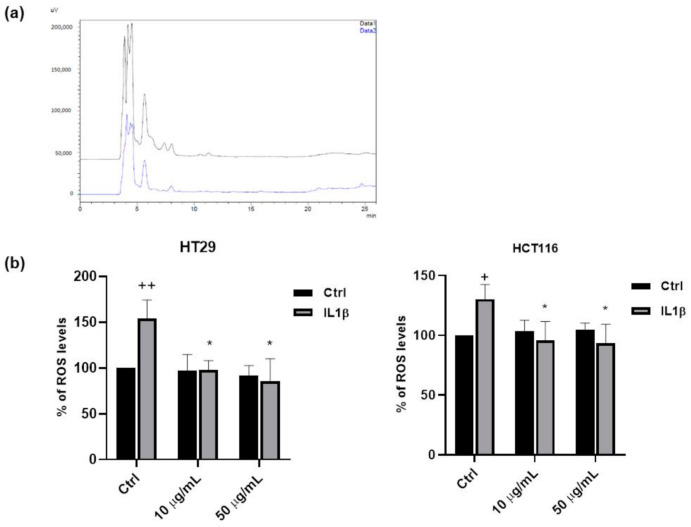

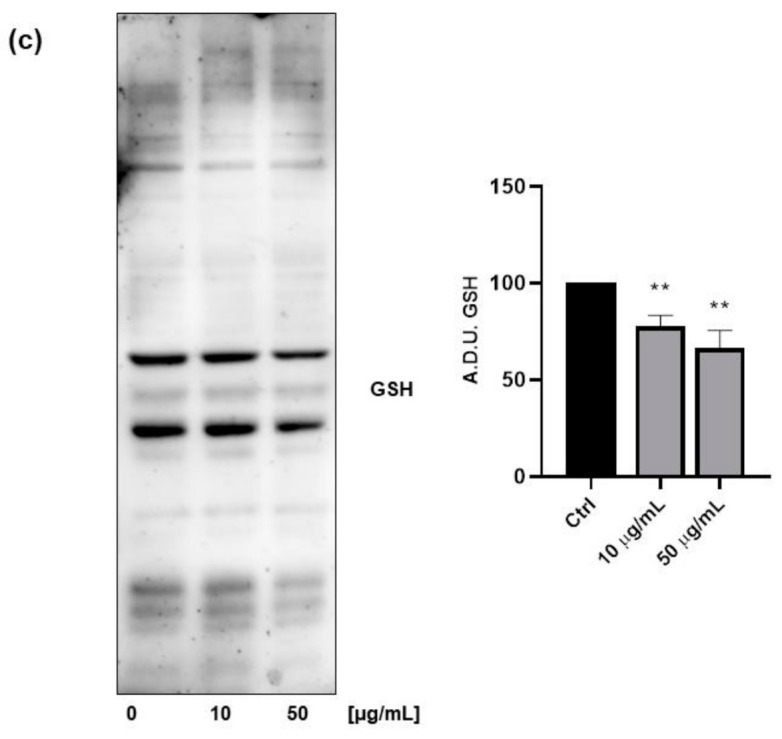
Antioxidant activity of FV extract. (**a**) HPLC-DAD-DPPH test. Chromatograms showed the differences in peak areas between the chromatograms obtained before (black) and after (blue) mixing DPPH and FV extract. (**b**) ROS measurement in HT29 and HCT116 cells after 18 h of exposure with the different concentrations of FV extract (10–50 µM) followed by a 24 h incubation with IL1β (10 ng/mL). Data are expressed as relative fluorescence units (++ *p* < 0.01 vs. Ctrl; + *p* < 0.05 *vs*. Ctrl; * *p* < 0.05 vs. IL1β). (**c**) Evaluation of the levels of glutathionylated proteins. HT29 cells were treated with different concentrations of FV extract (10–50 µM) for 36 h and then analyzed by western blot under non reducing conditions using an anti-GSH primary antibody. The gels are representative of three independent experiments (A.D.U.: arbitrary densitometry units). ** *p* < 0.01 vs. Ctrl.

As previously reported [3], extracts of common beans obtained from different Italian varieties of *P. vulgaris* possess antioxidant and antiradicalic properties. According to that, the FV extract showed antiradicalic capacity, monitored by DPPH reduction. The comparison between the HPLC-DAD chromatograms obtained before and after adding DPPH to FV extract showed that differences in peak areas related to polyphenols occur (Figure 3a), thus demonstrating that some constituents of FV extract were able to react with DPPH and underwent oxidative degradation.

Chlorogenic acid was the most degraded molecule, more than 75.0% after DPPH reaction, meaning that this hydroxycinnamic derivative primarily contributed to the antiradicalic activity of FV extract. The other main hydroxycinnamic derivative displayed a minor degradation (−39.4%); among phenolic acids, only gallic acid seemed to participate in DPPH reaction and its recorded degradation was 37.6%. Other polyphenolic constituents of FV extract did not show a significant degradation after DPPH reaction.

### 3.3. Antioxidant Properties of FV Extract

To further analyse the antioxidant properties of FV extract, we performed in vitro experiments on different cellular models. Due to the high nutraceutical impact and to the findings that common bean consumption is associated with in vivo chemoprotective effects at the early stages of colon cancer [16] and pro-apoptotic and anti-proliferative activities in vitro [14], we selected two different cellular models of colorectal adenocarcinoma, HT29 and HCT116 cells. To mimic a pro-oxidant and pro-inflammatory milieu, we stimulated colon cancer cells with interleukin 1β (IL1β, 10 ng/mL, 48 h) in the presence and in the absence of different concentrations of FV extract (10, 50 µg/mL), and we measured ROS levels by means of DCFH_2_-DA assay. As reported in Figure 3b, the ability of IL1β to promote ROS production was inhibited by the FV extract, at both concentrations and in both cell lines. It is well known that, in addition to triggering traditional post-translational protein modifications (including phosphorylation, acetylation, ubiquitination, etc.), ROS can directly modify cellular proteins, adding another layer of protein regulation to the proteome classified as oxidative post-translational modifications (OPTMs). In particular, ROS may cause various types of chemical modifications of proteins, including glutathionylation [25,26]. To explore the possibility that FV extract could affect these mechanisms, we treated colon cancer cells with the FV extract for 36 h and we measured the levels of glutathion–protein complexes by western blotting.

Figure 3c shows that the FV extract was able to significantly reduce the levels of total glutathionylated proteins, indicating the antioxidant activity of the FV extract. 

### 3.4. Anti-Inflammatory Properties of FV Extract

To further explore the biological properties of the FV extract, we investigated its ability to reduce the inflammation related to cancer. It is well known that inflammation is a key component in colon cancer onset and progression and that the cyclooxygenase 2 (COX-2) pathways play a major role in modulating cell growth, apoptosis and epithelial mesenchymal transition (EMT) [27,28,29]. Recent reports indicate that a direct interplay exists between inflammation and carcinogenesis. In fact, the risk of developing colon cancer is increased by chronic inflammatory diseases (such as inflammatory bowel disease), chronic infections or inflammations caused by environmental exposures. In addition, administration of COX inhibitors, such as aspirin and other non-steroidal, anti-inflammatory drugs (NSAIDs), is connected with a lower risk of developing colon cancer and its recurrence [30,31,32]

**Figure 4:**
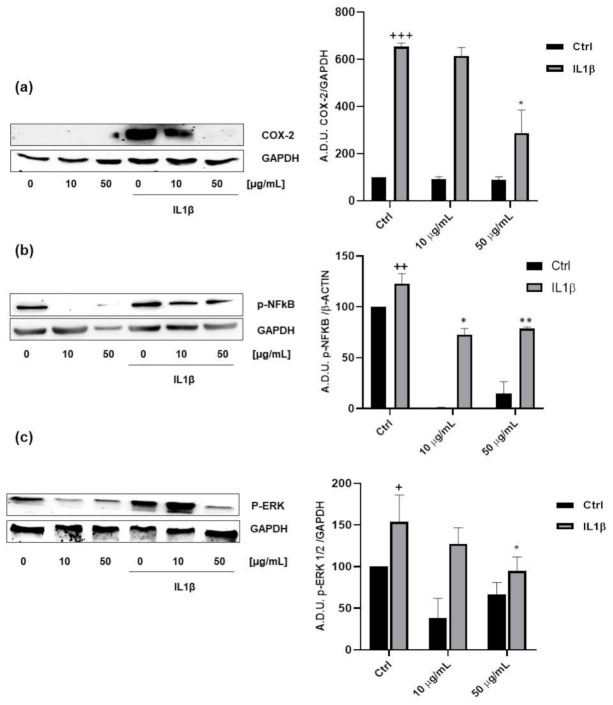
Anti-inflammatory activity of the FV extract. (**a**) Western blot analysis and quantification of COX-2 expression in HT29 cells after 18 h of exposure with different concentrations of FV extract (10–50 µM) followed by a 24 h incubation with IL 1β (10 ng/mL). +++ *p* < 0.001 vs. Ctrl, * *p* < 0.05 vs. IL1β. (**b**) Western blot analysis and quantification of NFκB phosphorylation in HT29 cells after exposure with FV extract (10–50 µM) followed by incubation with IL 1β (10 ng/mL). ++ *p* < 0.01 vs. Ctrl, **p < 0.01 vs. IL1β, * *p* < 0.05 vs. IL1β (**c**) Western blot analysis and quantification of ERK 1/2 phosphorylation in HT29 cells. (A.D.U.: arbitrary densitometry units). + *p* < 0.05 vs. Ctrl, * *p* < 0.05 vs. IL1β. The gels showed in the figure are representative of four independent experiments.

It has been demonstrated that IL1β, a pro-inflammatory cytokine, induces COX-2 expression in colorectal cells and that COX-2 drives colon cancer progression [33,34]. In this context, we evaluated the activity of FV extract on COX-2 expression induced by IL1β. We stimulated HT29 cells with IL1β (10 ng/mL, 48 h), in presence of 10 and 50 µg/mL of FV extract and we observed that at the higher concentration FV extract was able to strongly inhibit IL1β-induced COX-2 expression (Figure 4a). These data clearly indicate that FV extract could reduce the inflammation related to cancer.

It has been described that natural compounds, and in particular dietary polyphenols, exhibit a relevant anti-inflammatory activity linked to the inhibition of NFκB, MAPK and iNOS signalling [35]. In this light, we investigated the activity of the FV extract on NFkB and MAPK activation. By analysing the phosphorylation levels of NFkB and ERK1/2, we showed that the FV extract was able to reduce both p-NFkB and p-ERK1/2 levels in IL1β-stimulated colon cancer cells (Figure 4b,c), indicating once again an anti-inflammatory activity of FV components.

**Figure 5:**
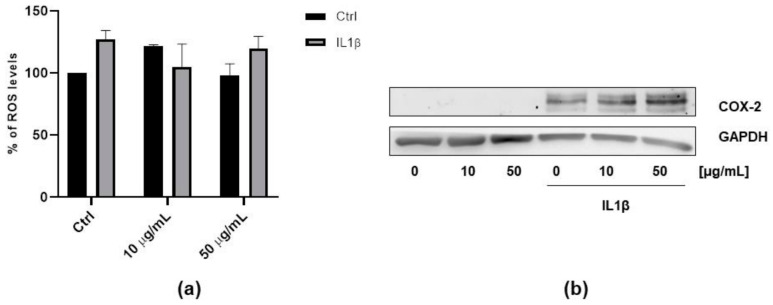
FV extract activity in endothelial cells. (**a**) ROS measurement in HUVEC cells after 18 h of exposure with the different concentrations of FV extract (10–50 µM) followed by a 24 h incubation with IL1β (10 ng/mL). Data are expressed as relative fluorescence units. (**b**) Western blot analysis of COX-2 expression in HUVEC cells after 18 h of exposure with different concentrations of FV extract (10–50 µM) followed by a 24 h incubation with IL 1β (10 ng/mL).

In order to further investigate the activity of FV extract, we measured the ROS levels and COX-2 expression in non-cancer cells. To this aim we used Human Umbilical Vein Endothelial Cells (HUVEC) as a model in which it is well known the activity of IL1β in inducing ROS production and COX-2 expression [36]. As reported in Figure 5, while IL1β treatment induced both ROS production and COX-2 expression, FV extract was shown to be inactive (Figure 5a,b), indicating a potential selective role of FV extract on cancer cells.

### 3.5. Anti-Proliferative Activity of FV Extract

**Figure 6:**
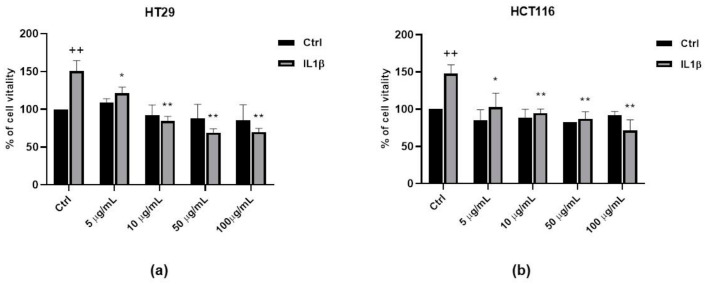
FV extract inhibits colon cancer cell growth. Cell proliferation induced by IL 1β (10 ng/mL) in the presence or absence of FV extract (5, 10, 50 and 100 µg/mL) was measured by MTT assay. HT29 (**a**) and HCT116 (**b**) cells were exposed to IL 1β for 48h. Data are reported as % of cell viability and are the means of 4 experiments run in triplicate. ++ *p* < 0.01 vs. Ctrl, * *p* < 0.05 and ** *p* < 0.01 vs. IL 1β.

In order to evaluate whether the FV extract could modulate tumor progression, we studied the proliferation of colon cancer cells by MTT test. HT29 and HCT116 were treated with 10 ng/mL IL1β for 48 h in the presence of increasing concentrations of the FV extract (from 5 to 100 µg/mL). According to previous studies conducted on white beans [3], the FV extract did not modify cancer cells’ growth in basal conditions. However, when the cells were exposed to an inflammatory milieu (IL1β), FV extract was able to reduce cell growth in a concentration-dependent manner (Figure 6a,b). Taken together, these data strongly support a potential biological activity of the FV extract, especially in inflammatory conditions.

## 4. Discussion

The identification of new species and varieties of vegetables is of fundamental importance to produce safe food and to obtain nutritional supplements and functional foods.

Food biodiversity has a high impact on public health and can offer more nutritious and healthier foods for rural and urban consumers, and provides opportunities to generate income and contribute to sustainable rural development. Therefore, there is an urgent need to develop and promote strategies for sustainable diets, mainstreaming biodiversity and nutrition as a common path, promoting nutrition-sensitive development and food-based approaches to solving health problems.

*Phaseolus vulgaris* is cultivated all over the world and is the most important edible legume for direct consumption. However, beans are more than a foodstuff since they are rich in many compounds with biological activity.

In the present work, we analysed for the first time the “Fagiola di Venanzio” (FV), a recently identified Italian variety of *P. vulgaris,* with the aim to characterize its chemical composition and potential biological activities. We initially analyzed four different samples of beans cultivated in four different areas within Murlo, a small municipality in the south of Tuscany. The areas of origin of the four samples are quite close to each other, but they differ in terms of soil composition and exposure. Nevertheless, the four samples did not demonstrate significant differences in chemical composition. We found that FV beans are rich in polyphenols including phenolic acids and hydroxycinnamic derivatives, as previously reported for other Italian varieties of *P. vulgaris.*

Since the beneficial effects of polyphenols on human health are expressed mainly through the reduction in oxidative stress, we evaluated the antioxidant activity of FV extract in in vitro assays and we reported that FV possesses antiradicalic activity *in vitro* and reduces ROS production promoted by interleukin 1β in two different models of colon cancer cells. In addition, the DPPH assay suggested that, in FV extract, chlorogenic acid primarily acts as a radicalic neutralizer. We also showed that the FV extract is able to reduce the expression of the inflammatory marker COX-2, the activation of NFkB and ERK1/2 MAPK and colon cancer cell growth promoted by IL1β, a well known pro-inflammatory cytokine. Interestingly, while FV extract did not inhibit cell growth in basal conditions, according to the results reported in the previous literature for white beans [3], we observed a strong reduction in proliferation induced by IL1β. It is well known that IL1β mimics inflammatory conditions that occur in intestinal tract and that may drive the development of such inflammatory chronic diseases as cancer, or such inflammatory bowel diseases (IBD) as ulcerative colitis and Crohn’s disease. Our results, showing that FV extract is able to reverse the effects of IL1β on colon cancer cells, strongly suggest that FV may play an important role in preventing the alteration of molecular processes characterizing the inflammatory microenvironment that leads to cancer and chronic diseases. In this scenario, these data suggest that bean consumption may be helpful in the prevention or treatment of inflammatory diseases of intestinal tract, and outline once again the importance of nutrition for human health.

## 5. Conclusions

In this work, we characterized the Fagiola di Venanzio, recently recognized and classified as a novel variety of *P. vulgaris* but never studied before, by determining its chemical composition and potential biological activity. This initial work on FV has been preparatory to a comparative work aimed at determining differences and similarities with other varieties of *P. vulgaris,* which is currently in progress. FV is cultivated in a very restricted area of a small municipality in the South of Tuscany, by a very restricted number of growers, and for this reason is in danger of extinction due to the spread of commercial varieties. As FV appears to be a promising source of bioactive compounds and rich in nutraceutical properties, more in-depth studies aimed to further elucidate its biological and nutraceutical potential will be fundamental to safeguard and promote this specific variety of *Phaseolus*, and to better understand the implications of its consumption for public health.

## Figures and Tables

**Table 1:** “Fagiola di Venanzio” samples

Grower	# Sample
Società Agricola Aiellino	1
Nicola Ulivieri	2
Burresi family	3
Azienda Agricola Podere Vignali	4

**Table 2:** Chemical composition of FV extracts

Components	Quantification (mg/g)			
	#1	#2	#3	#4
Total polyphenols	0.142 ± 0.018	0.123 ± 0.011	0.129 ± 0.016	0.120 ± 0.015
Total hydroxycinnamic derivatives	0.054 ± 0.004	0.051 ± 0.003	0.046 ± 0.005	0.052 ± 0.005
Isoflavones	<0.005	<0.005	<0.005	<0.005

**Table 3:** Chemical composition of #1 FV extract.

Composition	Quantification
Total polyphenols	0.131 ± 0.016 mg/g
Total hydroxycinnamic derivatives	0.046 ± 0.004 mg/g
Gallic acid	0.052 ± 0.005 mg/g
Chlorogenic acid	0.011 ± 0.002 mg/g
Isoflavones	<0.005 mg/g
Total soluble carbohydrates	10.032 ± 0.820 mg/g
Total proteins	15.190 ± 2.020 mg/g
